# A Novel MAO-B/SSAO Inhibitor Improves Multiple Aspects of Dystrophic Phenotype in *mdx* Mice

**DOI:** 10.3390/antiox13060622

**Published:** 2024-05-21

**Authors:** Francesca Gasparella, Leonardo Nogara, Elena Germinario, Lucia Tibaudo, Stefano Ciciliot, Giorgia Piccoli, Francisca Carolina Venegas, Francesca Fontana, Gabriele Sales, Daniele Sabbatini, Jonathan Foot, Wolfgang Jarolimek, Bert Blaauw, Marcella Canton, Libero Vitiello

**Affiliations:** 1 Department of Biology, University of Padova, 35131 Padova, Italy; francesca.gasparella@phd.unipd.it (F.G.); francesca.fontana.1@unipd.it (F.F.); gabriele.sales@unipd.it (G.S.); 2 Department of Biomedical Sciences, University of Padova, 35131 Padova, Italy; leonardo.nogara@unipd.it (L.N.); elena.germinario@unipd.it (E.G.); giorgia.piccoli@unipd.it (G.P.); francisca.venegas@unipd.it (F.C.V.); 3 Veneto Institute of Molecular Medicine (VIMM), 35129 Padova, Italy; stefano.ciciliot@unipv.it; 4 Department of Pharmaceutical Sciences, University of Padova, 35131 Padova, Italy; 5 Department of Molecular Medicine, University of Pavia, 27100 Pavia, Italy; 6 Fondazione Istituto di Ricerca Pediatrica Città della Speranza (IRP), 35127 Padova, Italy; 7 Department of Neurosciences, University of Padova, 35128 Padova, Italy; daniele.sabbatini@unipd.it; 8 Unit of Biostatistics, Epidemiology and Public Health, Department of Cardiac, Thoracic, Vascular Sciences, and Public Health, University of Padova, 35131 Padova, Italy; 9 Syntara Ltd., Sydney, NSW 2086, Australia; jonathan.foot@syntaratx.com.au (J.F.); wolfgang.jarolimek@syntaratx.com.au (W.J.)

**Keywords:** Duchenne muscular dystrophy, MAO-B, SSAO, oxidative stress, *mdx* mice, inflammation, osteopontin, fibrosis, heart, skeletal muscle

## Abstract

Duchenne muscular dystrophy (DMD) is one of the most frequent and severe childhood muscle diseases. Its pathophysiology is multifaceted and still incompletely understood, but we and others have previously shown that oxidative stress plays an important role. In particular, we have demonstrated that inhibition of mitochondrial monoamine oxidases could improve some functional and biohumoral markers of the pathology. In the present study we report the use of dystrophic *mdx* mice to evaluate the efficacy of a dual monoamine oxidase B (MAO-B)/semicarbazide-sensitive amine oxidase (SSAO) inhibitor, PXS-5131, in reducing inflammation and fibrosis and improving muscle function. We found that a one-month treatment starting at three months of age was able to decrease reactive oxygen species (ROS) production, fibrosis, and inflammatory infiltrate in the tibialis anterior (TA) and diaphragm muscles. Importantly, we also observed a marked improvement in the capacity of the gastrocnemius muscle to maintain its force when challenged with eccentric contractions. Upon performing a bulk RNA-seq analysis, PXS-5131 treatment affected the expression of genes involved in inflammatory processes and tissue remodeling. We also studied the effect of prolonged treatment in older dystrophic mice, and found that a three-month administration of PXS-5131 was able to greatly reduce the progression of fibrosis not only in the diaphragm but also in the heart. Taken together, these results suggest that PXS-5131 is an effective inhibitor of fibrosis and inflammation in dystrophic muscles, a finding that could open a new therapeutic avenue for DMD patients.

## 1. Introduction

Duchenne muscular dystrophy (DMD), one of the most severe forms of inherited muscular dystrophies, is an X-linked recessive disorder caused by mutations in the dystrophin gene. Lack of dystrophin, a structural sub-sarcolemmal protein, interrupts the mechanical connection between the sarcomeres and the extracellular matrix; this in turn triggers a complex series of pathological events, amongst which are continuous events of fibrous degeneration/regeneration coupled to chronic inflammation, eventually leading to muscle wasting and fibro-fatty replacement. At present there is no effective cure, and although palliative care has considerably improved life expectancy and quality of life, patients eventually succumb to cardiac respiratory failure, usually between the second and fourth decade of life [1]. Currently, the gold-standard treatment that can be applied to the vast majority of patients relies on steroid anti-inflammatory molecules and drugs supporting cardiac function, although a number of other molecules are also in clinical trials [2,3]. A few gene therapy treatments have also been approved for clinical use in recent years [4], but they are aimed at specific subsets of patients, bearing defined mutations and/or with defined clinical features, and the long-term benefits of such treatments are yet to be determined.

While the genetic bases of DMD have been long identified, the downstream pathogenic mechanisms are much less clear. DMD muscles are characterized by massive inflammatory infiltrates, as dystrophin deficiency leads to cell death and continuous release of toll-like receptor ligands, which over-activate the immune response [5]. Semicarbazide-sensitive amine oxidase (SSAO) and monoamine oxidase B (MAO-B) are two different amine oxidases that are involved in different biological pathways, including inflammation and oxidative stress. They both catalyze the oxidation of endogenous and exogenous amines, producing aldehydes, ammonia, and H_2_O_2_, a mediator of oxidative stress. SSAO, also named vascular adhesion protein-1 (VAP-1) or amine oxidase copper-containing 3 (AOC3), is stored in intracellular granules of endothelial cells and, upon inflammation, it moves to the cell surface where it mediates the extravasation cascade and trafficking of inflammatory cells [6]. Both inhibitors and antibodies against SSAO have been shown to provide beneficial effects in animal models of inflammation and fibrosis [7] and SSAO inhibitors have been progressed into clinical studies for a variety of indications, where both safety and efficacy have been shown [8,9].

MAO-B has long been identified as a key player in the pathophysiology of neurodegenerative diseases, like Parkinson’s disease (where MAO-B inhibitors have been clinically prescribed for over 30 years), and Alzheimer’s disease [10]. We have recently uncovered a novel role for MAO-B in innate immunity, showing that H_2_O_2_ produced by MAO-B in isolated macrophages sustains the activation of the NOD-, LRR- and pyrin domain-containing protein 3 (NLRP3) inflammasome [11]. We have also suggested that MAO inhibitors could have potential therapeutic benefits for dystrophic muscle, based on preclinical evidence in both patients’ cell cultures and in the *mdx* mouse model [12,13]. While there are various pieces of evidence suggesting that SSAO inhibitors may have therapeutic potential for reducing fibrosis in different organs [14,15,16,17], such an effect has not been reported for MAO-B-specific inhibitors. The potential therapeutic benefits of MAO-B inhibitors for dystrophic muscle are thought to be related to their ability to reduce oxidative stress and inflammation, which are key contributors to muscle damage and degeneration. Overall, while both SSAO and MAO-B inhibitors have potential therapeutic benefits for various diseases, they are not interchangeable and their effects on fibrosis in dystrophic muscle have not been investigated.

For these reasons, in this study we sought to investigate if treating *mdx* mice with a dual MAO-B/SSAO inhibitor, named PXS-5131, could ameliorate their dystrophic phenotype in terms of inflammation, fibrosis, and muscle function.

## 2. Materials and Methods

### 2.1. Chemicals

PXS-5131 was provided by Pharmaxis Ltd. (Sydney, NSW, Australia), which has recently described its design and synthesis [18]. Unless otherwise stated, all other chemicals used were purchased from Sigma, Merck Life Science, Milan, Italy.

### 2.2. Mice and PXS-5131 In Vivo Treatments

*Mdx* (C57BL/10ScSn-*Dmd^mdx^*/J) and syngeneic wild-type (C57BL/10ScSnJ) mice were purchased from Jackson Laboratories (Sacramento, CA, USA) and Charles River (Milan, Italy), respectively, and were housed and bred in the animal facility of the Vallisneri Biology Building at the University of Padova. Animals had free access to food and water and were kept at a 12 h day–night cycle; cages contained environmental enrichments and accommodated a maximum of 4 adult animals. All experiments were carried out in compliance with national and European bylaws and had been approved by the local Institutional Animal Care and Use Committee (IACUC) and by the Italian Ministry of Health (Auth. 321/2019PR). PXS-5131 (6 mg/kg/day) was administered orally, as a water-based solution sweetened with 15% sucralose for palatability. The dosage was chosen based on preliminary data obtained by Pharmaxis. At the end of the treatment, animals were first analyzed for force measurements and then sacrificed by cervical dislocation; muscles were then harvested and snap-frozen in isopentane pre-cooled in liquid nitrogen.

### 2.3. Muscle Functional Assessment

Muscle function in vivo was assessed for the gastrocnemius muscle, as described previously [19]. Briefly, mice were anesthetized with a mixture of tiletamine/zolazepam (40 mg/kg) and xylazine (7 mg/kg) and the foot was mounted on a 305B muscle lever system (Aurora Scientific, Aurora, ON, Canada). A lever arm of 2.1 mm was used for all groups, as no major differences in body weight between various groups was observed. Two electrodes were then placed on both sides of the sciatic nerve, while the common peroneal nerve was cut. The knee was blocked and an electrical stimulation was applied to the sciatic nerve, inducing the isometric plantar flexion of the foot. The force–frequency curve was obtained by stimulating at increasing frequencies (starting with a single depolarization up to 150 Hz). Force was then normalized to the weight of gastrocnemius and *plantaris* muscles, as an estimate of specific force. Eccentric contractions were performed by moving the foot backward at a velocity of 40 mm/s while the gastrocnemius was stimulated with a frequency sufficient to induce full tetanic fusion (100 Hz). Contractions were repeated once every 20 s to avoid inducing fatigue. Experimental data were analyzed using a self-compiled program in LabView.

For diaphragms, force development measurements were performed in vitro in a vertical muscle apparatus (Aurora Scientific 300B) filled with a Ringer solution containing 11 mM glucose and 30 µM d-tubocurarine, bubbled with 95% O_2_–5% CO_2_, at 30 °C and pH 7.2–7.4. Tissue strips from central tendon to ribs were dissected, mounted, and stretched to the optimal length; electrical stimulation was supplied by two parallel electrodes, delivering supramaximal pulses (0.5 ms duration) by a Grass S44 electronic stimulator through a stimulus isolation unit (Grass SIU5). Muscle response was recorded through an isometric force transducer (Grass FT03) connected to an AT-MIO 16AD acquisition card (National Instruments). Three strips were dissected for each diaphragm and the mean value was considered for each recorded parameter. At the end of the experiment, strips were weighed, and twitch and tetanic tensions normalized to the wet weight (specific tension, N g^−1^). Time to peak of the twitch (CT) and maximum rate of rise of tetanic tension (Vmax) were also measured.

### 2.4. Creatine Kinase Assay

Serum creatine kinase (CK) was measured using a dedicated assay kit (BioAssay Systems, Hayward, CA, USA), following the manufacturer’s instructions. Blood was collected by retro-orbital puncture from anesthetized animals, before performing the functional measurements. Plasma samples were separated by centrifugation at 2500× *g* for 10 min and were stored at −80 °C until use.

### 2.5. Histological Analyses

Cryosections (12 µm thick) from the diaphragm, tibialis anterior (TA), and heart were fixed in 2% paraformaldehyde (PFA) and then stained with Sirius red (Direct Red 80, Merck Life Science, Milan, Italy) 0.02% in saturated picric acid water solution; upon staining, glass slides were quickly rinsed in 15% acetic acid, dehydrated in an ethanol ladder followed by xylene, and then mounted with Eukitt medium (ORSAtec, Bobingen, Germany). Three to four sections, separated by at least 100 µm, were cut for each muscle. Upon staining, each section was photographed with a Leica DM6B microscope (Leica Microsystems, Wetzlar, Germany), using the acquisition/merge function of the LAS X software suite (version 3.7.6.25997—https://www.leica-microsystems.com/, accessed on 14 May 2024); with this function, partially overlapping fields acquired with a 10× lens were automatically acquired and assembled to generate a single image of the section. Images were then post-processed with GIMP software (GNU Image Manipulation Program, version 2.10.36—https://www.gimp.org/, accessed on 14 May 2024) to remove any artifacts from the sections, e.g., empty spaces, areas of tissue overlap; connective tissue belonging to the epimysium (or epicardium) was also discounted. The relative areas of muscle fibers, stained in yellow, and of collagen-containing extracellular matrix, stained in red, were then measured using an in-house-designed macro for the FIJI software (Fiji Is Just ImageJ, version 2.14.0/1.54f—https://imagej.net/software/fiji/, accessed on 14 May 2024) [20]. For the morphological analyses, gastrocnemius cryosections were stained with DAPI plus wheat germ agglutinin (WGA, Thermo Fisher, W11262, Monza, Italy) conjugated with the Alexa Fluor^®^ 594 fluorophore, and then photographed with a Leica DM6B epifluorescence microscope with a 10× lens. Images were then analyzed with an in-house algorithm based on the sequential use of the SMASH MATLAB (MATLAB version R2023b (23.2)—https://www.mathworks.com, accessed on 14 May 2024) [21] and FIJI software packages.

### 2.6. Immunofluorescence Analyses

Immunofluorescence was performed on diaphragm and TA cryosections to assess neutrophil and macrophage inflammatory infiltrates, using anti-Ly6G and anti-F4/80 rat monoclonal antibodies. Briefly, slides were fixed in 2% PFA for 15 min, rinsed in PBS, saturated with anti-mouse IgG (Jackson ImmunoResearch, 115-007-003, Cambridge, UK) diluted 1:25 in 3% BSA for 1 h, followed by 45′ in 10% goat serum in PBS. Slides were then incubated at 4 °C overnight with either anti-Ly6G (Abcam, ab25377, Milan, Italy), 1:100 in BSA 3% in PBS, or anti-F4/80 (Abcam, ab6640), 1:75 in 3% BSA in PBS. Sections were then washed with PBS, incubated with Alexa Fluor^®^ 488 AffiniPure Donkey Anti-Rat IgG (H+L) (Jackson ImmunoResearch, 712-545-150) diluted 1:300 in BSA 3% in PBS at room temperature for 1 h, washed with PBS, and mounted with Fluoromount-G^™^ Mounting Medium with DAPI (Thermo Fisher).

At least five randomly chosen images of each section (3 to 4 sections per muscle) were acquired with a Leica DM6B epifluorescence microscope with a 20× lens. Images were then analyzed with GIMP software to determine the percentage of pixels exhibiting a fluorescent signal above background; the latter was determined in sections processed in the same session without the use of primary antibodies.

### 2.7. Dihydroethidium (DHE) Staining

Freshly cut diaphragm and TA cryosections were incubated within 30 min with 5 µM DHE (Thermo Fisher, D11347) for 30′ at 37 °C in degassed PBS + 2 mM EDTA, washed twice in PBS, and mounted with 10% glycerol. Images were then acquired within 24 h with a Leica DMI6000B inverted microscope (Leica Microsystems), from 3–4 randomly chosen fields per section, using a 20× lens and 568 + 25 nm excitation and 585 nm long-pass emission filters. Nuclear fluorescence data were then acquired and analyzed using Metamorph software (version 7.8—https://www.moleculardevices.com/, accessed on 14 May 2024) as previously described [12].

### 2.8. Transcriptomic Analyses

Total muscle transcriptomic analyses were performed on gastrocnemii muscles, one per animal, from a sub-cohort of nine 4-month-old mice: three untreated *mdx*, three PXS-treated *mdx* mice, and three aged-matched wild-type CD57BL/10ScSn mice. The nine muscles were sent to BGI Genomics Co., Ltd. (Hong Kong, China), where they were processed for total RNA extraction followed by ribosomal RNA depletion, cDNA synthesis, and sequencing. The sequencing was carried out with BGI’s proprietary DNBSEQ platform. Raw data were then downloaded from BGI’s cloud services and analyzed in house. Briefly, raw reads were trimmed to remove adapter sequences using cutadapt (version 4.0—cutadapt.readthedocs.io, accessed on 14 May 2024). The abundances of all mouse transcripts annotated by ENSEMBL (release 105) were estimated using the Salmon software (version 1.8.0—https://combine-lab.github.io/salmon/, accessed on 14 May 2024) [22] and then summarized at the gene level using tximport (version 1.22.0—https://bioconductor.org/, accessed on 14 May 2024) [23]. We excluded sample “K1” from the rest of the analysis due to the low mappability rate of its reads. Sequence reads are available on the NCBI BioProject database with the accession numbers PRJNA1079072. Genes were then filtered by their expression levels using the strategy described by Chen and colleagues [24] as implemented in the edgeR package. A total of 12,963 genes were retained.

Gene-level counts were normalized for GC-content and for unwanted variation using EDASeq (version 2.28.0—https://bioconductor.org/, accessed on 14 May 2024) and RUVSeq (version 1.28.0—https://bioconductor.org, accessed on 14 May 2024; RUVg method, k = 2 confounding factors) [25]. Differential expression was tested with edgeR (version 3.36.0—https://bioconductor.org, accessed on 14 May 2024) [26] using a GLM model. Genes with an adjusted *p*-value (FDR) < 0.05 after correction for multiple testing (Benjamini–Hochberg method) were considered differentially expressed.

Instant clue software (version 0.9.2) [27] was used to visually analyze transcriptome data; after being filtered by FDR as specified in the results, log transformed data were z-score normalized and then the hierarchical clustering was represented with a heatmap. Finally, enrichment analyses were carried out through NIH’s Metascape webtool [28].

### 2.9. Quantitative Real-Time Polymerase Chain Analyses

Total RNA was isolated from gastrocnemius muscle cryosections with TRIzol (Invitrogen) according to the manufacturer’s instructions. Complementary DNA (cDNA) was obtained with random primers and a high-capacity cDNA reverse transcription kit (Thermo Fisher). Quantitative polymerase chain reaction (PCR) was performed with specific primers using the SYBR green PCR master mix (Applied Biosystems, Monza, Italy) in an ABI PRISM 7500 real-time PCR system (Applied Biosystems).

Primer sequences were as follows: *Spp1*: fwd-GCTTGGCTTATGGACTGAGGTC rev-CCTTAGACTCACCGCTCTTCATG; *Gapdh*: fwd-GCAAAGTGGAGATTGTTGCCAT rev-CCTTGACTGTGCCGTTGAATTT.

Relative expression data were calculated with the 2^−ΔΔCt^ method, using the RNA extracted from wild-type muscles as a calibrator and the *Gapdh* gene as normalizer.

### 2.10. Statistical Analyses

Statistical analyses were performed with the Prism 9 software (GraphPad, Boston, MA, USA); data were log-transformed before using the appropriate parametric tests (*t*-test or ANOVA). When multiple measures for the same muscle were present, i.e., for all histological analyses, the “nested analyses for repeated measures” tests were used.

## 3. Results

### 3.1. Inhibition of SSAO and MAO-B Decreased Oxidative Stress, Inflammatory Infiltrate and Collagen Deposition in mdx Mice

**Figure 1:**
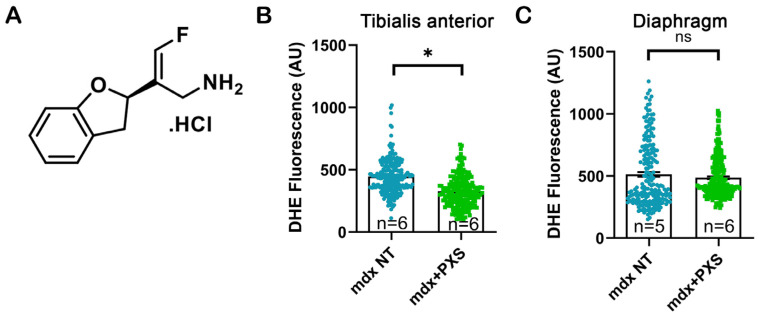
(**A**) Molecular structure of PXS-5131. (**B**,**C**) DHE fluorescence quantification from tibialis anterior muscle sections and diaphragm muscle sections, respectively, in untreated (*mdx* NT, blue dots) and treated (*mdx*+PXS, green dots) mice. Each chart point represents the fluorescence measured in a single nucleus; statistical analysis was performed via nested *t*-test, considering the single muscles as independent biological replicates. n indicates number of animals; ns: not significant, * *p*-value < 0.05.

PXS-5131 (Figure 1A) is a nanomolar irreversible inhibitor of SSAO and MAO-B with good selectivity over the related amine oxidases and no oxidative turnover. It is a mechanism-based inhibitor of both enzymes, affording long lasting inhibition from low daily dosing [29].

In order to understand if dual inhibition of SSAO and MAO-B could ameliorate muscle pathology in *mdx* mice, we treated 3-month-old adult animals with PXS-5131 (6 mg/kg/day) for one month. Upon sacrificing, the effect of PXS-5131 treatment on oxidative stress in skeletal muscles was assessed using the DHE fluorescent dye. When oxidized, DHE turns into ethidium, which can then intercalate into genomic DNA and increase its emission levels. Quantification of the nuclear fluorescence signal showed a significant reduction in treated animals for the TA, but not in the diaphragm (Figure 1B,C).

**Figure 2:**
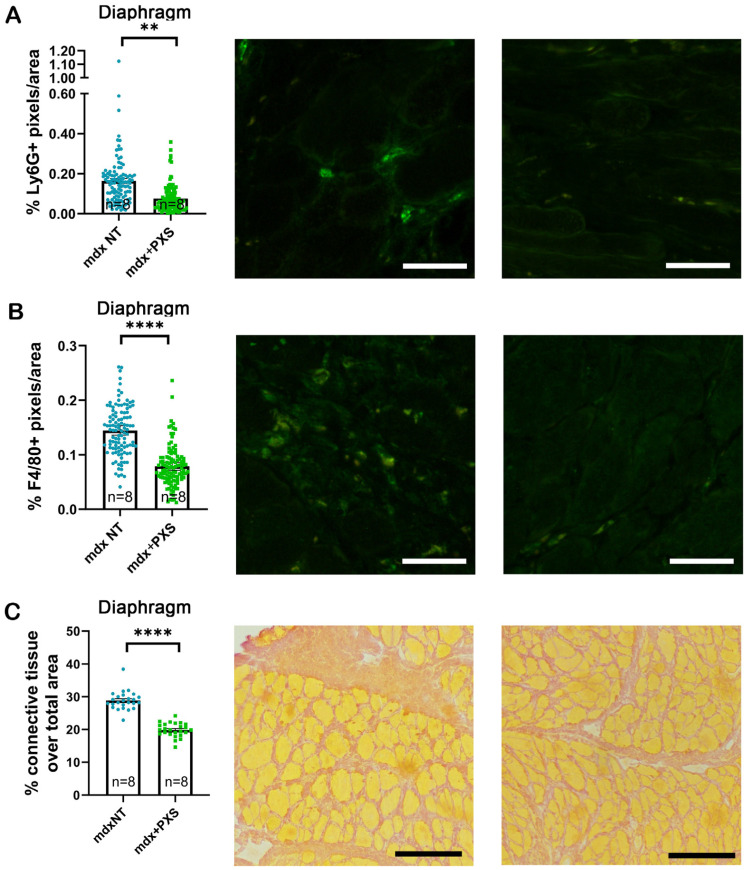
(**A**,**B**) Charts to the left show the quantification of neutrophils (expressed as Ly6G-positive pixels over total area) and macrophages (expressed as F4/80-positive pixels over total area) from diaphragm sections of untreated (*mdx* NT, blue dots) and treated (*mdx*+PXS, green dots) mice. Each chart point represents the output of a single microscopy field; statistical analysis was performed via nested t-test, considering the single muscles as independent biological replicates. n indicates number of animals; ** *p*-value < 0.01, **** *p*-value < 0.0001. Immunofluorescence images show examples of the antibody staining as seen in magnified areas of fields acquired from a control (center) and a treated animal (right). The scale bars correspond to 70 microns. (**C**) Quantification and representative images of Sirius red staining on diaphragm sections of 4-month-old *mdx* mice. Each chart point represents the measurement obtained from a whole tissue section; statistical analysis was performed via nested *t*-test, considering the single muscles as independent biological replicates. n indicates number of animals; **** *p*-value < 0.0001. Brightfield images show magnified areas from a control (center) and a treated (right) animal. The scale bars correspond to 140 microns.

SSAO and MAO-B inhibition and reduction in oxidative stress are known to affect the inflammatory response. For this reason, we assessed the amount of infiltrating neutrophils, identified as Ly-6G+, and the amount of macrophages, identified as F4/80+, in the muscles of treated and untreated animals. As can be seen in Figure 2A,B, there was a significant reduction in the amount of both cell types in the diaphragms of treated mice and a similar decrease was also found in the TA muscles (Supplementary Figure S1A,B). Next, we examined the amount of fibrosis present in the muscles of treated and control *mdx* mice by means of Sirius red staining. The extent of collagen-rich (red-stained), fibrotic areas was greatly reduced in diaphragms from treated animals (Figure 2C). Once again, analyses of TA muscles showed a similar trend, although the overall amount of fibrotic areas was about half of that found in the diaphragm (Supplementary Figure S1C).

### 3.2. Treatment with PXS-5131 Partially Prevented Force Drop upon Eccentric Contractions

Besides histological analyses, we also carried out multiple force measurements aimed at determining if the inhibition of MAO-B and SSAO could positively affect the contractile properties of dystrophic muscle. To this aim, we used a set of in vivo and ex vivo measurements that had been previously optimized by our group. In particular, torque production of the plantar flexors was determined after electrical stimulation.

**Figure 3:**
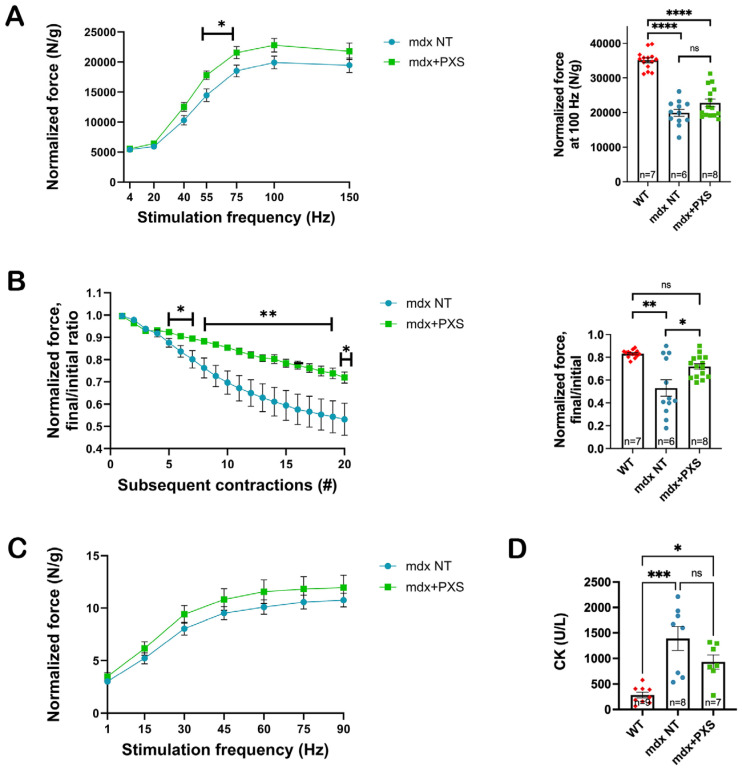
(**A**) Left-hand chart shows the normalized force–frequency relationship in the plantar flexors in untreated (*mdx* NT, blue dots) and treated (*mdx*+PXS, green dots) mice. Each data point represents the average value for each frequency; statistical analysis was performed with an unpaired *t*-test for each frequency. Right-hand chart shows the details of the tetanic force measured with a stimulation at 100 Hz. As a reference, the force expressed by a set of aged-matching wild-type animals (WT, red dots) is also shown. Each data point represents the output of a single leg; statistical analysis was performed via unpaired *t*-test. n indicates number of animals; ns: not significant; * *p*-value < 0.05, **** *p*-value < 0.0001. (**B**) Left-hand chart shows the decline in force production after repeated eccentric contractions of the *plantar flexors*. Each data point represents the average value for each contraction; statistical analysis was performed with an unpaired *t*-test for each contraction. Right-hand chart shows the detailed relative force decline after 20 eccentric contractions. As a reference, the effect of the same experimental protocol in aged-matching wild-type animals is also shown. Each data point represents the output of a single leg; statistical analysis was performed via unpaired *t*-test. n indicates number of animals; ns: not significant; * *p*-value < 0.05, ** *p*-value < 0.01. (**C**) Force–frequency relationship measured ex vivo on diaphragm strips (three for each animal). Each data point represents the average value for each frequency; statistical analysis was performed via nested *t*-test, considering the single muscles as independent biological replicates. The maximum force measured in diaphragms from age-matched wild-type animals was between 18 and 20 N/g (not shown). (**D**) Creatine kinase (CK) concentrations assessed in the plasma of wild-type and untreated and treated *mdx* mice. The difference between treated and untreated mice did not reach statistical significance (*p* = 0.13). Each data point represents an animal; statistical analysis was performed via one-way ANOVA followed by Tukey’s multiple comparisons test. n indicates number of animals; ns: not significant; * *p*-value < 0.05, *** *p*-value < 0.001.

As can be seen in Figure 3A, there were no major changes in force production normalized for muscle weight after one month of treatment with PXS-5131, except for some significant increases at stimulation frequencies below those leading to a complete tetanic stimulation. While showing a reduction in normalized tension, a major functional deficit observed in dystrophic *mdx* mice is the loss in force production after repeated lengthening contractions [19]. Indeed, in our hands, control *mdx* mice lost about 45% of maximal force production after 20 eccentric contractions, whereas PXS-treated mice only lost 23% (Figure 3B), in line with previous results showing that inhibition of MAO-B activity alone also had a protective effect against eccentric contraction [13]. After completing the force analyses, gastrocnemius muscles were cryosectioned and analyzed with histological techniques in order to evaluate the possible effect of PXS-5131 on muscle regenerative processes, by measuring the fibers’ cross-sectional area (CSA) and percentage of central nuclei. As shown in Supplementary Figure S2, even though there was a positive trend in both cases, i.e., fewer small and centrally nucleated fibers in treated mice, neither analyses reached statistical significance.

We then proceeded to analyze the contractile properties of diaphragms, by examining ex vivo the force produced by tissue strips taken from animals immediately after sacrifice. While normalized force was always greater in treated versus control mice at all frequencies, the differences did not reach statistical significance (Figure 3C). Lastly, as activity-related muscle damage leads to an increase in circulating muscle proteins, like troponin I or creatine kinase, we examined CK levels in wild-type, treated, and control *mdx* mice. We found a trend towards a decrease in CK levels in treated mice, in line with the results obtained from the eccentric contraction measurements, but the high variability found between different animals, including amongst wild-type animals, did not allow these results to reach statistical significance (Figure 3D).

### 3.3. Effects of PXS-5131 Treatment on Gene Expressions

**Figure 4:**
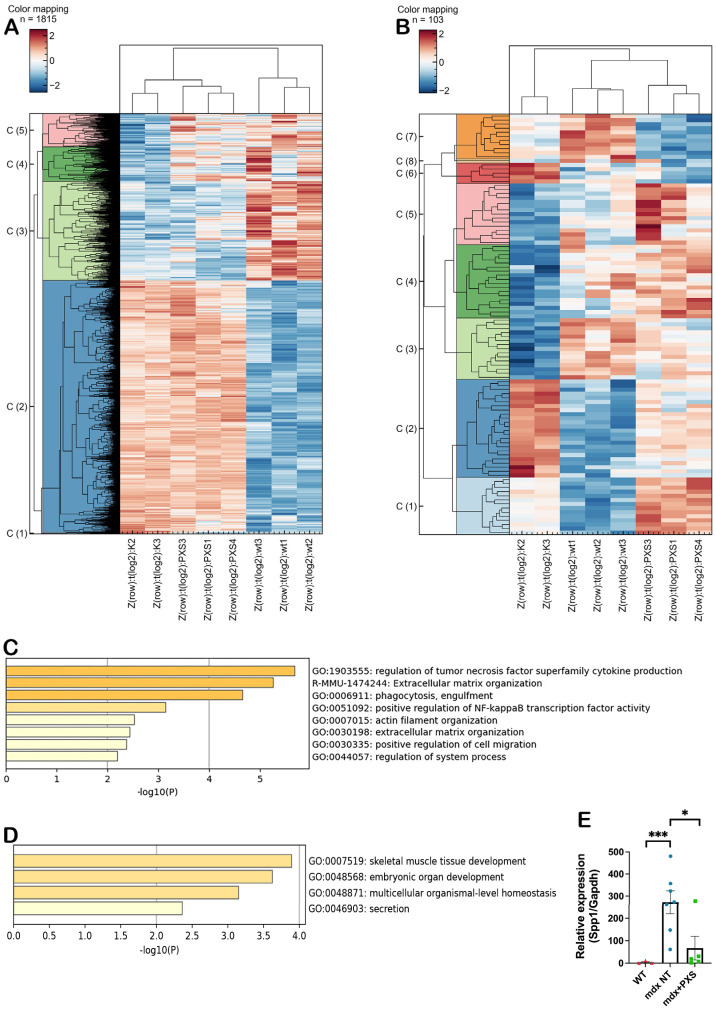
(**A**) Heatmap showing Euclidean hierarchical clustering of Z-score normalized, log transformed gene expression of differentially regulated transcripts, comparing untreated *mdx* and wild-type mice (FDR < 0.05). Chart shows the relative gene expression of untreated (K1 and K2) and treated (PXS1, PXS3, PXS4) *mdx* and wild-type (wt1, wt2, wt3) mice. (**B**) Heatmap showing Euclidean hierarchical clustering of Z-score normalized, log transformed gene expression of differentially regulated transcripts of untreated *mdx* vs. wild-type mice (FDR < 0.05) as well as treated vs. untreated *mdx* mice (FDR < 0.1). Chart shows the relative gene expression of untreated (K1 and K2) and treated (PXS1, PXS3, PXS4) *mdx* and wild-type (wt1, wt2, wt3) mice. (**C**) Metascape analysis of C2 cluster from panel B; bar graph shows enriched terms in the genes exhibiting a down-regulation after treatment with PXS-5131. (**D**) Metascape analysis of C3 cluster from panel B; bar graph shows enriched terms in the genes exhibiting a down-regulation after treatment with PXS-5131. (**E**) Results of real-time PCR analysis of *Spp1* transcript levels in gastrocnemius muscles from treated (green dots) and untreated (blue dots) *mdx* mice (n = 5–7) and wild-type animals (red dots; n = 3). Statistical analysis was performed via one-way ANOVA on log-transformed values, followed by Tukey’s multiple comparisons test. n indicates number of animals; * *p*-value < 0.05, *** *p*-value < 0.001.

To try and get some mechanistic insight into the observed histological and functional improvements after treatment with PXS-5131, we performed an RNA-sequencing analysis on gastrocnemius muscles from wild-type and *mdx* mice, either untreated or treated with PXS-5131. In order to understand if and how the dystrophic transcriptome was altered by the treatment, we determined the z-score-normalized expression values of the 1815 genes that showed a significant alteration (FDR < 0.05) in untreated *mdx* mice compared to wild-type animals (Supplementary Table S1), and plotted them using a hierarchical clustering. As can be seen from Figure 4A, the gene expression profile in treated *mdx* mice was overall similar to that of untreated controls.

Next, we searched for differentially expressed genes (DEGs) between controls and PXS-5131-treated animals; using a threshold of FDR < 0.1 we identified 103 DEGs, 63 upregulated and 40 downregulated. These genes were once again subjected to hierarchical clustering, which produced eight distinct clusters (Figure 4B and Supplementary Table S2). Of these, clusters C2 to C5 contained genes whose expression in treated animals was changed towards the levels found in wild-type mice. Each group of genes was then analyzed using the “Metascape” bio-informatic tool. Cluster C2 was identified as involved primarily in inflammatory processes and extracellular matrix organization (Figure 4C), whereas cluster C3 was identified as involved primarily in skeletal muscle tissue development and developmental and homeostatic processes (Figure 4D); clusters C4 and C5 did not contain any category that could be directly linked to muscle pathology. Intriguingly, one of the most downregulated genes in the C2 cluster was osteopontin (*Spp1*), a key regulatory player of fibrosis in DMD muscle [29]. This latter finding was then confirmed via quantitative real-time PCR, performed on total RNA extracted from the gastrocnemius muscles, as it was the case for the samples used in the RNA-seq (Figure 4E). Given the clear reduction in macrophages and neutrophiles found in PXS-treated mice, we also verified if this was reflected in the expression of the inflammatory cytokines Interleukin 1-beta (IL1-β), Interleukin 6 (IL6) and Tumor Necrosis Factor-alpha (TNF-α). Indeed, the analysis showed that PXS-5131 treatment had led to a marked reduction for all three transcripts, which was statistically significant for IL1-β and IL6 (Supplementary Figure S3).

### 3.4. Longer PXS-5131 Treatment Decreased Histological Alterations in the Diaphragm and Heart at Later in Older Mice

**Figure 5:**
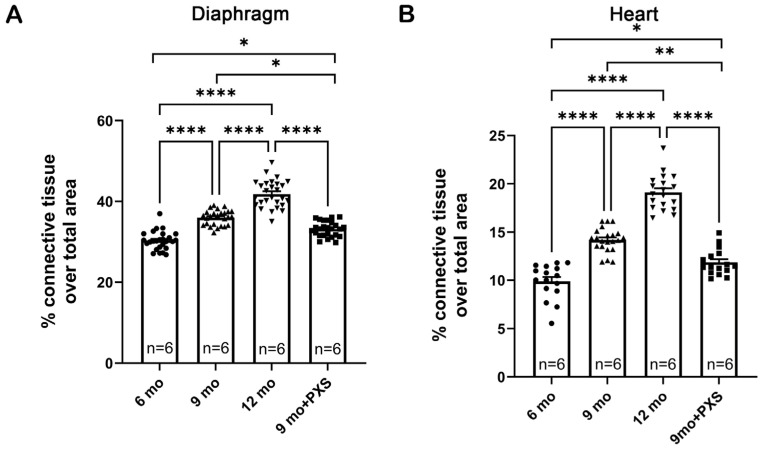
Quantification of fibrosis from diaphragm muscle sections (**A**) and heart sections (**B**) in 6-, 9-, and 12-month-old untreated and treated *mdx* mice. Both muscles had been stained with Sirius red. Animals were treated starting at age 6 months. Each data point represents the measurement obtained from a whole tissue section; statistical analysis was performed via nested t-test, considering the single muscles as independent biological replicates. n indicates number of animals; * *p*-value < 0.05, ** *p*-value < 0.01, **** *p*-value < 0.0001.

In *mdx* mice cardiac muscle also exhibits progressive fibrotic remodeling, but such a phenomenon appears later in life compared to skeletal muscle. As one month of treatment showed a significant decrease in fibrosis, we decided to determine if administering PXS-5131 over a longer time period could lead to beneficial effects not only in the diaphragm but also in the heart. As shown in Figure 5A, the fibrotic area in the diaphragm muscle steadily increased over time, when comparing 6-, 9-, and 12-month-old *mdx* mice; the same trend was also found in the ventricular myocardium (Figure 5B and Figure S4). Importantly, a 3-month-long treatment with PXS-5131 starting at six months of age was able to significantly reduce such increases in both muscles.

## 4. Discussion

Duchenne muscular dystrophy is characterized by repeated cycles of degeneration and regeneration of muscle fibers, accompanied by a strong inflammatory response, oxidative stress, and progressive fibro-fatty remodeling, leading to a progressive impairment in skeletal muscle function. Similar changes occur in the heart, even though these alterations occur at later stages than in skeletal muscle, both in mice and in humans. In this manuscript we show that treatment of dystrophic *mdx* mice with a dual MAO-B/SSAO inhibitor for one month reduced oxidative stress in the tibialis anterior, as well as fibrosis and infiltration of neutrophils and macrophages in the tibialis anterior and in the diaphragm. In addition, a three-month treatment of older *mdx* mice was able to significantly prevent the deposition of fibrosis in the myocardium. Besides these histological changes, we also observed a reduced sensitivity to damage induced by lengthening contractions in the gastrocnemius muscles. These histological and functional improvements, however, did not seem to originate from a major remodeling of the dystrophic transcriptome, although specific genes linked to extracellular matrix remodeling were significantly downregulated after treatment with PXS-5131.

It is well established that inflammation is a key player in DMD; however, its precise role(s) in its pathophysiology is not fully elucidated. The functional improvements observed after treatment with corticosteroids in DMD boys suggest an important role for the immune system in affecting muscle function. Indeed, the negative role played by pro-inflammatory macrophages in the observed pathological features is clear from multiple studies [30,31]. One study showed that antibody-dependent depletion of macrophages was sufficient to reduce membrane permeability in 4-week-old *mdx* muscles by 80% [32]. While not measured in this study, a reduced muscle membrane instability with less macrophage infiltration would fit well with the reduction in damage due to lengthening contractions observed in this study, as this is generally considered to be due to increased membrane permeability [33]. Indeed, repeated eccentric contractions are known to lead to an increase in membrane permeability, leading to a calcium overload, increased oxidative stress, and subsequent muscle dysfunction [19,34,35]. In addition to a reduction in total macrophage levels, a shift from pro- to anti-inflammatory macrophages has in itself a moderating effect on the formation of fibrosis in dystrophic muscle [31,36], ameliorating muscle function. This suggests that part of the improvements we observe here after treatment of PXS-5131 is likely due to the reduction in macrophagic infiltrate.

PXS-5131 treatment also led to a decrease in the number of neutrophils in the muscle tissue. The importance of neutrophil infiltration in dystrophic muscle has been shown by the fact that treatment with anti-neutrophil antibodies significantly ameliorated muscle damage [37]. Also, recent results showed how a murine cathelicidin-related antimicrobial peptide (Cramp), which has chemotactic effects on neutrophil recruitment during muscle damage, was strongly increased in dystrophic muscle compared to control animals. Loss of Cramp reduces the presence of Ly6G-positive neutrophils in dystrophic muscle and significantly reduces the fibrosis, as measured by Sirius red staining [38]. Interestingly, one of the mechanisms leading to these beneficial effects passes through the modulation of Sarco-Endoplasmic Reticulum Calcium ATPase (SERCA) activity, linking inflammation to intracellular calcium homeostasis, which is a well-known modulator of muscle physiology and its increase is sufficient to induce a muscular pathology [39].

We have previously shown that inhibition of both isoforms of mitochondrial monoamine oxidase (MAO-A and B) could improve the dystrophic phenotype by reducing oxidative stress in two murine models of muscular dystrophies [12]. Later studies carried out with safinamide, a MAO-B inhibitor already used in the clinical setting for Parkinson’s disease [40], suggested that the reduced oxidative stress could also protect against activity-dependent muscle damage [13]. Here we show that treatment with PXS-5131 also decreased oxidative stress, although the effect was present in tibialis anterior muscles but not in diaphragms, while fibrosis and inflammatory infiltrates were reduced in both muscles. The different effect exerted by PXS-5131 on reactive oxygen species (ROS) levels in the diaphragm and tibialis anterior was somewhat unexpected; such findings could be due to different levels of ROS production and/or to different roles played by MAO-B and SSAO in the two muscles. In this regard it is worth noting that both activity levels and fiber types are known to affect the levels of oxidative stress [41], as it is also suggested by the fiber type-specific effects on muscle contractility in wild-type and dystrophic muscles [42]. A similar muscle fiber-specific response was seen in selenoprotein N KO mice, in which an increased oxidative stress in the sarcoplasmic reticulum specifically affected the diaphragm, but not the hindlimb muscles [43,44].

In terms of mechanism(s) of action, it is also worth noticing that neither MAO-B nor SSAO expression seems to be upregulated in *mdx* muscles when compared to wild-type controls, as shown by our own transcriptome data (MAO-B, logFC = 0.3 FDR = 0.51; SSAO/AOC3, logFC = −0.64 FDR = 0.32) and published literature [45,46]. Thus, we hypothesize that an increased availability of substrates for these enzymes is responsible of the higher production of H_2_O_2_.

In this work we did not carry out a direct comparison between PXS-5131 and safinamide; however, it is worth noticing that while the latter is a fairly non-specific compound (safinamide also inhibits glutamate release and dopamine and serotonin reuptake, it binds to the sigma receptors, and it can block sodium and calcium channels [47]), PXS-5131 is highly selective for SSAO and MAO-B. Our present findings hence are a further confirmation that MAO-B is definitely a relevant target in the context of DMD treatment.

Improvement in support therapies for DMD, such as the introduction of nocturnal assisted ventilation, has greatly prolonged patients’ life expectancies [48]. This in turn has led to a steady increase in the prevalence of DMD-related cardiomyopathy, which at present is amongst the main causes of mortality [48]. In the *mdx* model, however, the cardiac phenotype is much less prominent than in patients, similarly to what happens for skeletal muscle, and it becomes apparent later in life [49]. For this reason, in this study we also looked at the fibrosis in the heart of older *mdx* mice, at six, nine, and twelve months of age, and verified the effects of a 3-month long PXS-5131 treatment starting at six months. As expected, during the six- to twelve-month time frame there was a marked increase in the fibrotic process that mirrored, albeit with lower percentages, that seen in the diaphragm of the same animals. Importantly, PXS-5131 administration was able to reduce the progression of cardiac fibrosis by ~17%, a percentage that was actually higher than that found in the diaphragms (~8%). The fact that the treatment was able to continue to exert a positive effect also later in life, when the dystrophic phenotype was more pronounced, suggests that a chronic treatment started early in life might be a useful therapeutic option.

Perhaps not surprisingly, given that PXS-5131 was not expected to directly act at the transcriptional level, when performing an RNA sequencing analysis comparing *mdx* mice treated with PXS-5131 for one month with untreated animals, we found that the overall transcriptome in the former was only marginally different from that of untreated controls. However, bio-informatic analyses of the DEGs identified four gene clusters in which wild-type and *mdx* mice were at the opposite sides of the expression spectrum and PXS-5131 had clearly moved treated mice towards the former. In particular, two of the four clusters appeared to be directly relevant for the dystrophic phenotype, as a Metascape analysis tagged one as involved in the organization of the extracellular matrix and the other in skeletal muscle development. Of note, the first DEG cluster included the osteopontin gene (*Spp1*), a known modifier gene in DMD [50,51]. Polymorphisms in the promoter region of *Spp1* are linked to more pronounced disease progression and loss of ambulation in patients. In *mdx* muscles it is expressed at higher levels in regenerating fibers and infiltrating cells [52] and it has recently been identified as a key regulator of fibrosis via a specific macrophagic population [53]. The reduction we observed in *Spp1* expression likely reflects, at least in part, the reduction in inflammatory infiltrate in skeletal muscle. As alterations in osteopontin levels are also linked to the prevention of activity-dependent damage and loss of ambulation [50], it is tempting to speculate that it could play a role in the protective effect we observed after eccentric contractions. In terms of the possible connection(s) between PXS-5131 and *Spp1* levels, it should be noticed that osteopontin upregulation is also found in conditions other than DMD, such as atherosclerosis and hypertension [54], and it appears to be H_2_O_2_-dependent [55]. It is also known that H_2_O_2_ induces the expression of several proteins through the activation of ROS-sensitive transcription factors [56]. In the case of osteopontin, H_2_O_2_-dependent transcription was found to be mediated by increased binding of both activator protein 1 (AP-1) and nuclear factor kappa-light-chain-enhancer of activated B cells (NF-kB), specifically to the −2284 to −795 region of the OPN promoter, with a subsequent increase in mRNA and protein levels [55]. As we recently showed that in inflammatory conditions MAO-B-driven H_2_O_2_ production over-activates NF-κB [11,57], we hypothesize the existence of a MAO-B/NF-κB/osteopontin axis. In this regard, it is also possible to hypothesize a feedforward cycle, as it has been reported that in cancer cells osteopontin induces NF-κB activation [58].

## 5. Study Limitations and Conclusions

Overall, our results show that the treatment of adult, *mdx* dystrophic mice with a new MAO-B/SSAO dual inhibitor for one month is sufficient to reduce oxidative stress and inflammation, attenuate muscle fibrosis, and improve the resistance to lengthening contractions. In older mice, extended treatment reduces the progression of fibrosis in both the diaphragm and heart. These results indicate that inhibition of both SSAO and MAO-B may be beneficial in the treatment of DMD, and highlight the potential of the dual inhibitor PXS-5131.

As this is the first instance of PXS-5131 being used in the context of muscular dystrophy, this study presents some limitations that will have to be addressed with further research. At this time, our data do not allow us to address the mechanisms behind the differential effect of the drug on the diaphragm and tibialis anterior. In this regard, one could envisage the use of different experimental approaches, e.g., oxyblots and/or metabolomic analyses. Another aspect that will need to be further explored is the specific influence of PXS-5131 on the composition of the inflammatory infiltrate in terms of myeloid and lymphoid cell sub-populations, via FACS analyses. By the same token, performing the transcriptomic analyses on specific cell sub-populations, including single fibers, could also shed more light on the pathways affected by the treatment. Last but not least, the effects of longer treatments on disease progression will need to be assessed both at the level of skeletal and cardiac muscle.

## Data Availability

Sequencing data have been posted in the NCBI BioProject database with the accession number PRJNA1079072.

## Supplementary Materials

The following supporting information can be downloaded at: https://www.mdpi.com/article/10.3390/antiox13060622/s1, Figure S1: Treatment of 3-month-old *mdx* mice with PXS-5131 for one month is sufficient to reduce inflammation and fibrosis in tibialis anterior muscle. Figure S2: Treatment with PXS-5131 did not significantly alter muscle fiber morphology. Figure S3: Treatment with PXS-5131 decreased the expression of inflammatory cytokines at the RNA level. Figure S4: Representative images of fibrosis progression in the heart of *mdx* mice at different ages. Table S1: z-score-normalized expression values of DEGs between untreated *mdx* mice and wild-type animals. Table S2: z-score-normalized expression values of DEGs resulting from the combined analyses of *mdx* vs. wild-type and *mdx* controls vs. treated *mdx*.
