# Supplementary material for: A Novel MAO-B/SSAO Inhibitor Improves Multiple Aspects of Dystrophic Phenotype in mdx Mice
Source: Antioxidants (Basel). 2024 May 21;13(6):622. doi: 10.3390/antiox13060622 (PMC11201281; doi:10.3390/antiox13060622)
Supplement: Supplementary file 1 [file antioxidants-13-00622-s001.zip › Supplementary figures.pdf]

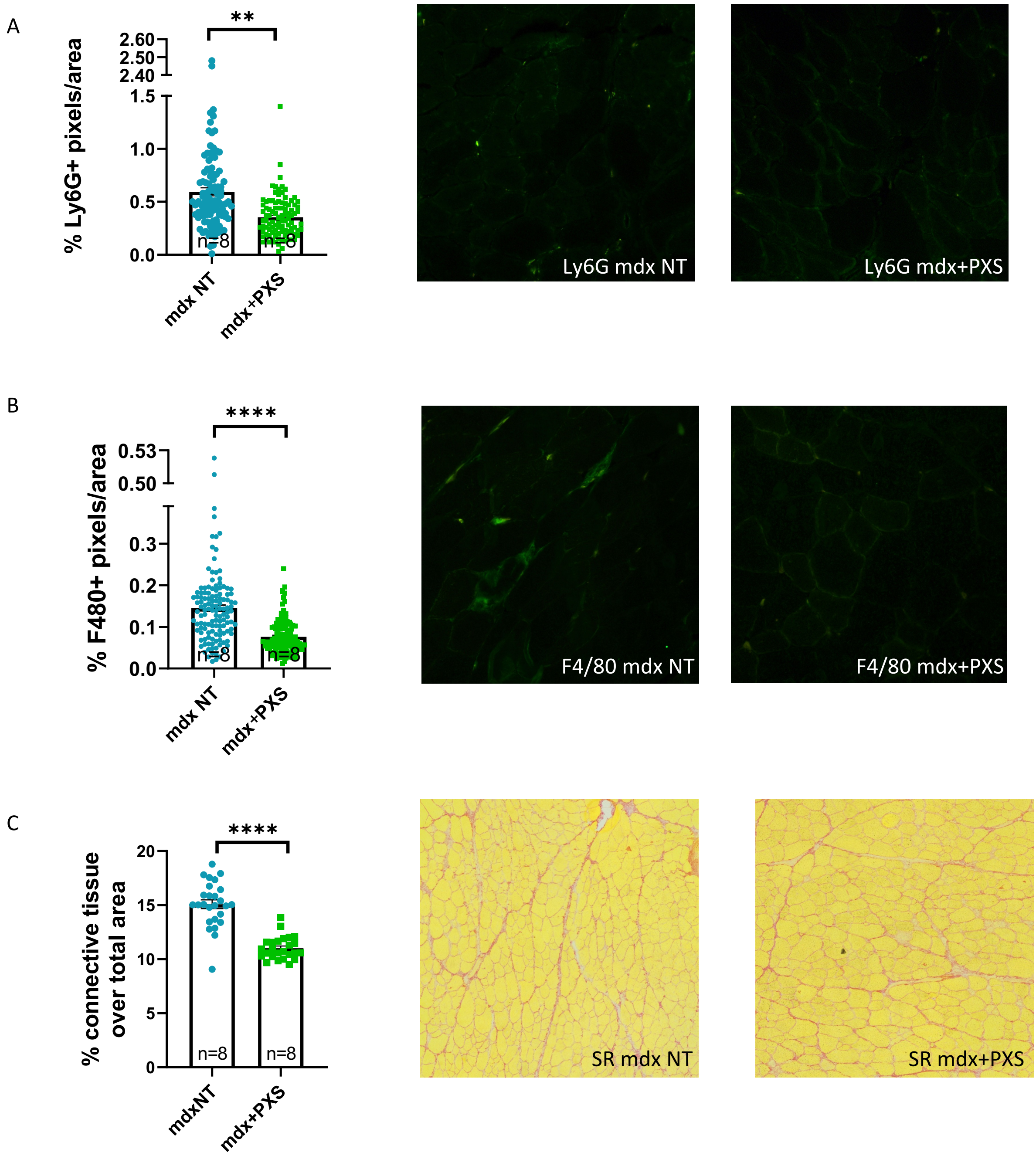
A
**
2.60
2.50
2.40
1.5
1.0
0.5
0.0
% Ly6G+ pixels/area
n=8
n=8
mdx NT
mdx+PXS
Ly6G mdx NT
Ly6G mdx+PXS
B
****
0.53
0.50
0.3
0.2
0.1
0.0
% F480+ pixels/area
n=8
n=8
mdx NT
mdx+PXS
F4/80 mdx NT
F4/80 mdx+PXS
C
****
20
15
10
5
0
% connective tissue over total area
n=8
n=8
mdxNT
mdx+PXS
SR mdx NT
SR mdx+PXS

Figure S1: Treatment of 3-month-old mdx mice with PXS-5131 for one month is sufficient to reduce inflammation and fibrosis in Tibialis Anterior muscle.

A) and **B)** Charts to the left show the quantification of neutrophils (expressed as Ly6G-positive pixels over total area) and macrophages (expressed as F4/80-positive pixels over total area) from tibialis anterior sections of 4-month-old *mdx* mice. Each chart point represents the output of a single microscopy field; statistical analysis was performed via nested t-test, considering the single muscles as independent biological replicates. n indicates number of animals; ** p-value < 0.01, **** p-value < 0.0001. Immunofluorescence images show examples of the antibody staining as seen in magnified areas of fields acquired from a control (center) and a treated animal (right). Scale bars corresponds to 70 microns. **C)** Quantification and representative images of Sirius red staining on tibialis anterior sections of 4-month-old *mdx* mice. Each chart point represents the measurement obtained from a whole tissue section; statistical analysis was performed via nested t-test, considering the single muscles as independent biological replicates. n indicates number of animals; **** p-value < 0.0001. Brightfield images show magnified areas from a control (center) and a treated (right) animal. Scale bars corresponds to 140 microns.

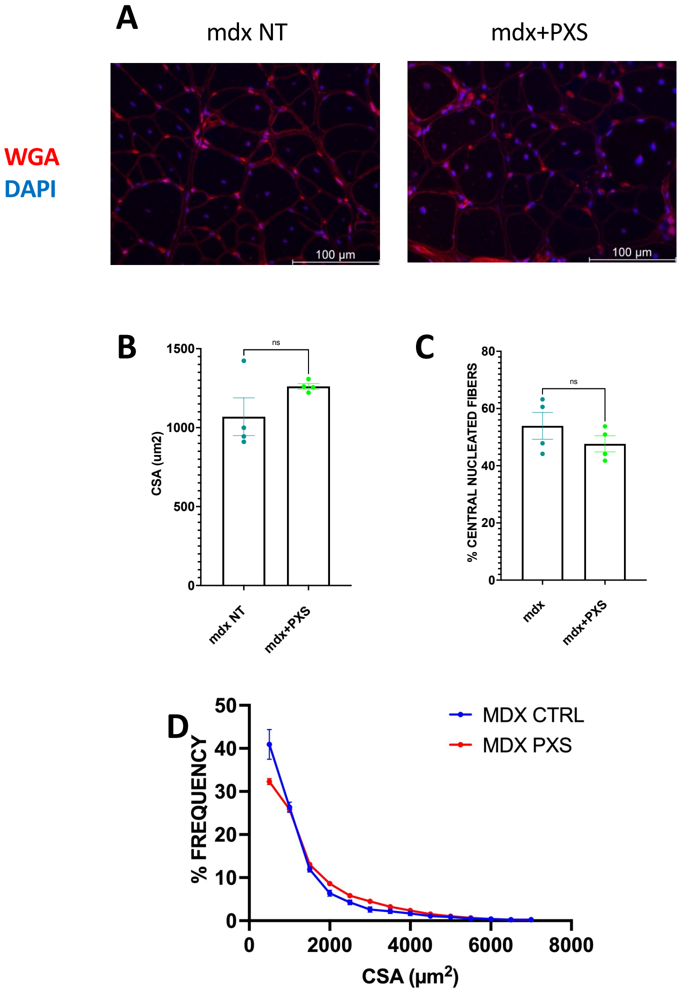

Figure S2: Treatment with PXS-5131 did not significantly alter muscle fibers morphology.

A) Representative gastrocnemius sections stained with DAPI (blue) and wheat germ agglutinin, WGA (red). **B)** Mean cross-sectional area (CSA) of gastrocnemius muscle fibers shows no differences between non-treated and PXS treated *mdx* mice (n=4 muscles per group; 4000-6000 fibers per muscle). **C)** Analysis of central nuclei shows no significant differences in the percentage of central nucleated fibers between the two groups of mdx mice (n=4 per group; 2000-2500 fibers). Data are shown as mean ± SEM. Statistical analysis was performed using two-tailed Student's t-test. **D)** Chart shows the CSA distribution of all the fibers used to calculate the averages shown in panel B.

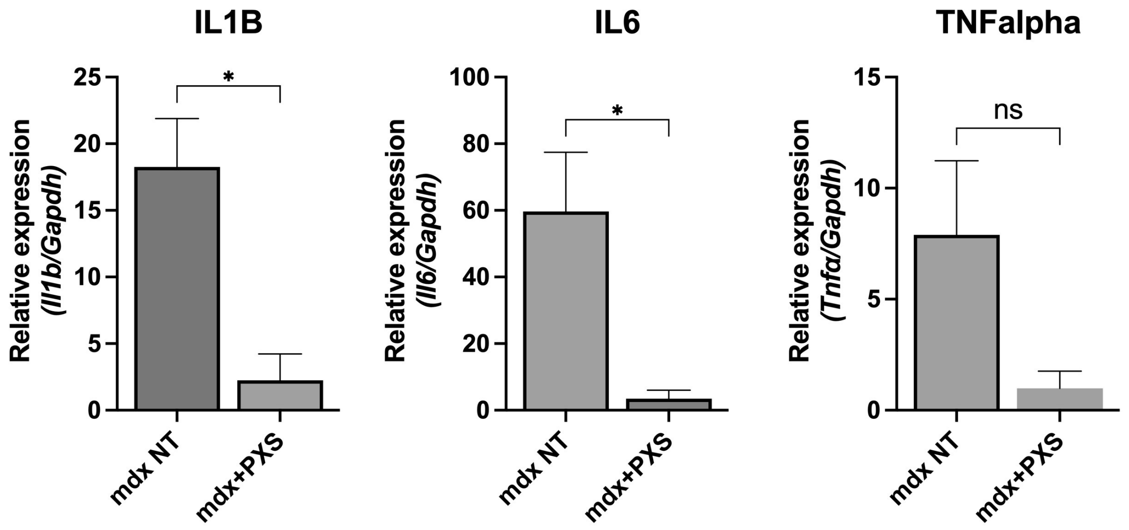

Primer sequences:

Il6: fwd-AGGATACCACTCCCAACAGAC rev-GCCATTGCACAACTCTTTTCTC

Il1β: fwd-GGACATGAGCACCTTCTTTTCC rev-TTGTTCATCTCGGAGCCTGTAG

Tnf: fwd-GAAAAGCAAGCAGCCAACCA rev-CGGATCATGCTTTCTGTGCTC

Figure S3: Treatment with PXS-5131 decreased the expression of inflammatory cytokines at the RNA level.

Real-time PCR were performed on the same samples used for Spp1 quantification, following the same experimental procedure. Given the low levels of cytokine expression, however, some samples were excluded and numerosity was at least ≥3.

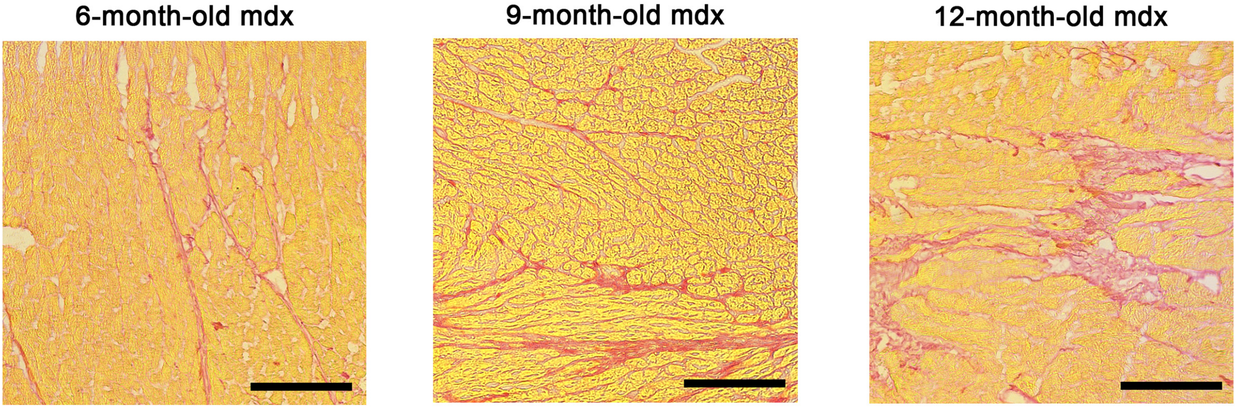

Figure S4: Representative images of fibrosis progression in the heart of *mdx* mice at different ages.

Heart cryosections from *mdx* mice were stained with Sirius Red; the age of the animals is reported on top of each panel. Scale bars corresponds to 140 microns.
